# Experimental Investigation and Analytical Modeling of Chloride Diffusivity of Fly Ash Concrete

**DOI:** 10.3390/ma13040862

**Published:** 2020-02-14

**Authors:** Jian Zhang, Xin-Zhu Zhou, Jian-Jun Zheng, Hai-Long Ye, Jin Yang

**Affiliations:** 1 Jiyang College, Zhejiang Agriculture and Forestry University, Zhuji 311800, China; JianZhang_zjyc@163.com (J.Z.); JinYang_jyc@163.com (J.Y.); 2 School of Civil Engineering and Architecture, Zhejiang University of Technology, Hangzhou 310023, China; jjzheng@zjut.edu.cn; 3 Department of Civil Engineering, The University of Hong Kong, Pokfulam Road, Hong Kong, China; hlye@hku.hk

**Keywords:** fly ash concrete, chloride diffusivity, experimental investigation, analytical method

## Abstract

Owing to its importance in the assessment of reinforced concrete structures, it is essential to determine the chloride diffusivity of fly ash concrete. This paper presents an investigation into the diffusion characteristics of chloride ions in fly ash concrete. Through experiment, the relationship between chloride diffusivity and curing age up to 1800 days is measured and the effects of curing age, water/binder ratio, aggregate volume fraction, and fly ash content (i.e., percentage of total cementitious material by mass) on chloride diffusivity are evaluated. It is found that the chloride diffusivity decreases with the increase of curing age, aggregate volume fraction, and fly ash content, but increases with the increase of water/binder ratio. In analytical modeling, an equivalent aggregate model is constructed and the equivalent interfacial transition zone (ITZ) thickness is derived analytically. With the equivalent aggregate model, three-phase fly ash concrete reduces to a two-phase composite material. By extending the Maxwell method, the chloride diffusivity of fly ash concrete is formulated. Finally, the validity of the analytical method is verified by experimental results.

## 1. Introduction

Reinforced concrete structures may be subjected to reinforcement corrosion when they are built in a harsh environment. As a result, some harmful species, such as chloride ions and sulfate ions, penetrate into concrete under the concentration gradient and cause corrosion of reinforcing steel bars and concrete cracking. Therefore, the chloride diffusivity of concrete plays an important role in assessing the durability of reinforced concrete structures [1,2].

To improve the durability of reinforced concrete structures, fly ash is often added into concrete as a mineral admixture, and the chloride diffusivity can be reduced significantly owing to the pozzolanic reaction of fly ash [3,4]. It has been shown that the pozzolanic reaction of fly ash occurs mainly in later curing ages from 90 to 360 days [5,6,7]. Such hydration characteristics can reduce the porosity and improve the pore structure of concrete for a long time [8,9], therefore making concrete increasingly denser and improving the penetration resistance against chloride ions. Poon et al. [5] demonstrated by a mercury-injection method that mixing fly ash into mortar and net cement paste can greatly reduce the porosity of the interfacial transition zone (ITZ) and the cement paste matrix. Ahmaruzzaman [10] further confirmed that the accumulation effect posed by fly ash particles on the aggregate surface and the pozzolanic reaction can make the ITZ disappear eventually.

Much effort has been made to determine the chloride diffusivity of concrete experimentally and theoretically. Thomas et al. [11] observed from an in-situ test that, within a curing time of 28 days, the decreasing effect of fly ash incorporation on the chloride diffusivity of concrete is negligibly small. As the curing age increases, however, the decreasing effect becomes more and more significant. Zhou et al. [12] studied the relationship between chloride diffusivity of concrete and fly ash content (i.e., percentage of total cementitious material by mass) in an electrical conductivity test and concluded that adding fly ash into cement paste significantly reduces the chloride diffusivity. Oh and Jang used a non-steady-state migration test to measure the chloride diffusivity of concrete and found that, when fly ash content is less than 25%, the chloride diffusivity decreases alongside an increase in fly ash content [13]. Otherwise, the opposite trend was observed. Yu and Ye [14] further studied experimentally the influence of curing age on the diffusion of chloride ions in fly ash concrete. They found from mercury intrusion porosimetry (MIP) tests that the microstructure is continuously improved during the curing period and fly ash still plays a part in enhancing the penetration resistance against chloride ions within a long duration. Moradllo et al. [15] adopted transmission X-ray microscopy and X-ray computed microtomography to investigate the in-situ diffusion of chloride ions in cement paste. Chalee et al. [16] took the water/binder ratio and the fly ash content as the experimental variables to analyze the long-term transport properties of concrete. Based on the test results, an empirical relationship was regressed between chloride diffusivity and curing age. In terms of theory, Damrongwiriyanupap et al. [17] formulated the chloride diffusivity of cement paste in terms of the porosity and the chloride diffusivity of pore solution and verified the analytical relationship with experimental results. Gu et al. [18] adopted a multi-scale model to compute the chloride diffusivity of ultra-high performance concrete and demonstrated that the numerical results slightly overestimate the experimental results. With a multi-level diffusion model, Ren et al. [19] proposed a hierarchy process for the effective prediction of the chloride diffusivity of manufactured sand mortar and to evaluate the influence of aggregate shape and limestone powder on chloride diffusivity. Based on a random walk algorithm, the chloride diffusivity of concrete was estimated [20]. Oh and Jang [13] quantified the effects of aggregate volume fraction and ITZ volume fraction on the chloride diffusivity of concrete by combining numerical methods with analytical approaches. Zheng et al. [21] proposed a differential effective medium approach to evaluate the effect of aggregate shape on the chloride diffusivity of concrete. Liu et al. [22] developed a numerical method to model the ingress of chloride ions into reconstructed concrete. Zhou et al. [12] presented an analytical approach to the chloride diffusivity of fly ash cement paste. By analyzing a short-term chloride exposure test, Zhang et al. [23] indicated that the power function can be used to describe the time-dependence of chloride diffusivity of concrete. For concrete with mineral admixtures, it is also appropriate to describe the relationship between chloride diffusivity and curing age with the power function [16]. The literature review above clearly shows that there are two limitations to the current studies. First, most experiments focus on the short-term transport properties of fly ash cement paste or concrete. Second, few theoretical models are available for estimating the chloride diffusivity of fly ash concrete. Therefore, it is necessary to investigate the long-term transport properties of fly ash concrete experimentally and theoretically.

The objective of this paper is to study the diffusion characteristics of fly ash concrete. An experiment is conducted to analyze the time dependence of chloride diffusivity and to evaluate the key influential factors. An analytical method is then presented to estimate the chloride diffusivity of fly ash concrete. Finally, the validity of the analytical method is verified with experimental results.

## 2. Chloride Diffusion Test of Fly Ash Concrete

### 2.1. Materials and Methods

This chloride diffusion test was conducted from September 2013 to December 2018. The fly ash concrete specimens were made with P.O 42.5 cement and fly ash of grade II [24]. Their chemical compositions are listed in Table 1. The apparent density and specific surface were 2300 kg/m^3^ and 295 m^2^/kg for the fly ash and 3150 kg/m^3^ and 342 m^2^/kg for the cement, respectively. Crushed stone and river sand were selected for the coarse aggregate and fine aggregate, respectively. The aggregate gradation obeyed the Fuller curve and the aggregate size was divided into seven grades, i.e., 0.15–0.3, 0.3–0.6, 0.6–1.18, 1.18–2.36, 2.36–4.75, 4.75–9.5, and 9.5–16 mm. The apparent density, water content, and water absorption were 2760 kg/m^3^, 0.48%, and 0.72% for the crushed stone and 2600 kg/m^3^, 0.58%, and 0.77% for the river sand, respectively. Tap water was used for mixing fly ash concrete, and a saturated Ca(OH)_2_ solution with pH of about 12.5 was adopted for curing the specimens to avoid the calcium leaching of fly ash concrete.

The water/binder ratio (w/b) of fly ash concrete was selected as 0.4 and 0.5. For each water/binder ratio, the aggregate volume fraction C_a_ was 0.55, 0.65, and 0.75 and the fly ash content m_f_ was 0.1, 0.2, and 0.3. The mix proportions of fly ash concrete are shown in Table 2.

**Figure 1:**
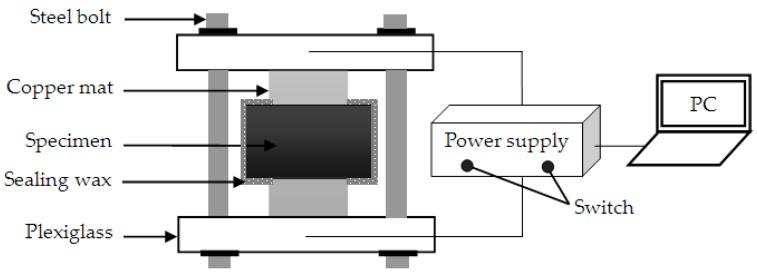
Schematic of test device.

The curing age t was set as 28, 60, 120, 240, 540, 720, 1260, and 1800 days. Concrete cylinders with dimensions of Φ 100 mm × 100 mm were cast. To obtain a representative mean, three identical cylinders were made for a given mix proportion and age. After 28 days of standard curing, concrete specimens with sizes of Φ 100 mm × 50 mm were formed by cutting the two edges of the concrete cylinders and again immersed in the saturated Ca(OH)_2_ solution at a curing temperature of 20 ± 2 °C. During the curing period, the saturated Ca(OH)_2_ solution was replaced monthly. Finally, the chloride diffusivity D_cf_ was measured by the electrical conductivity method [25]. The schematic of the test device is shown in Figure 1.

### 2.2. Experimental Results and Discussions

**Figure 2:**
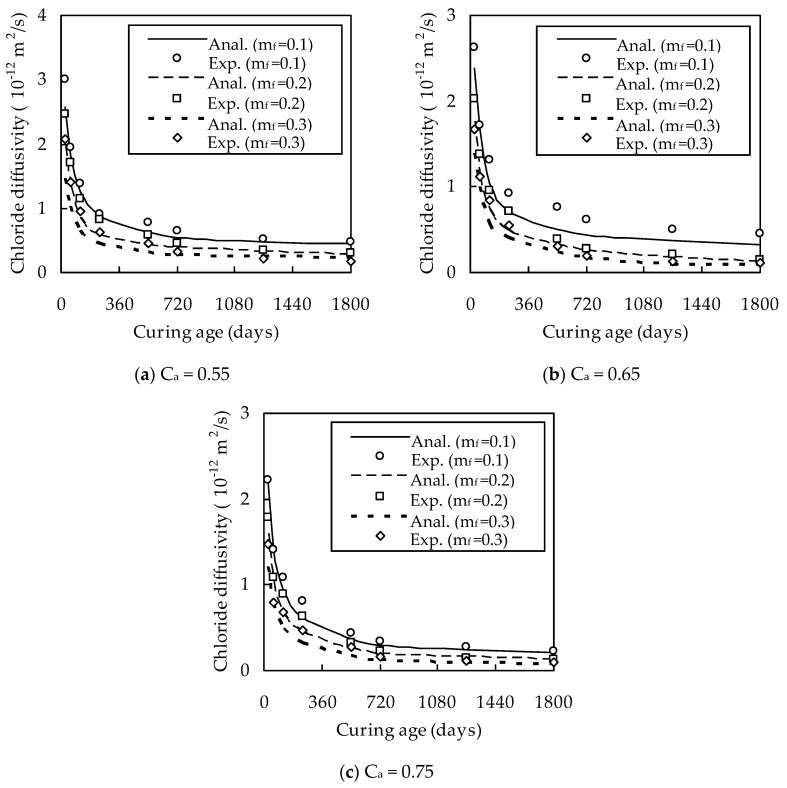
Variation of D_cf_ with t for w/b = 0.4.

**Figure 3:**
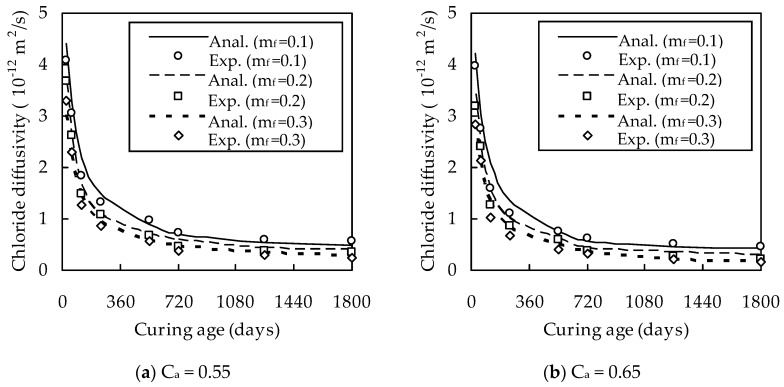

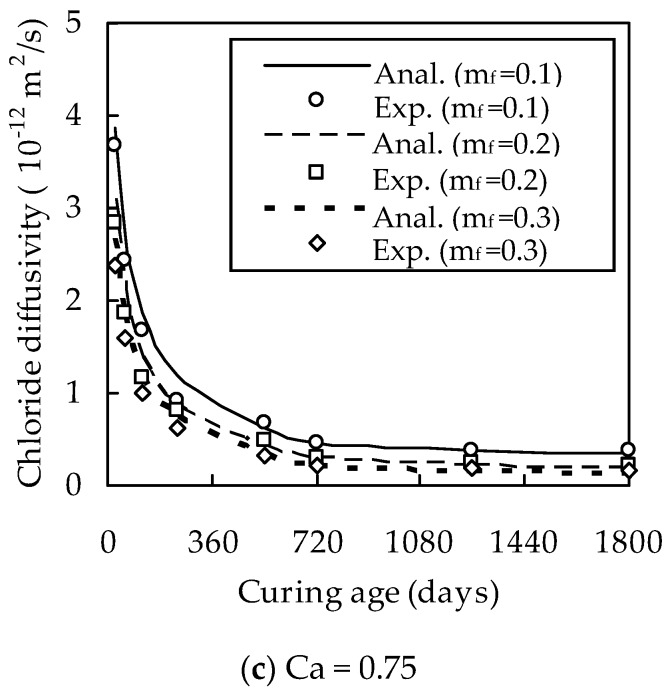
Variation of D_cf_ with t for w/b = 0.5.

The effects of w/b, C_a_, m_f_, and t on D_cf_ are shown in Figure 2 and Figure 3. It can be seen from Figure 2 and Figure 3 that D_cf_ increases with the increase of w/b. The reason for this is that a larger water/binder ratio results in a larger porosity. D_cf_ decreases almost linearly with increasing C_a_ for a given w/b, C_a_, and t owing to the dilute and tortuous effects of aggregates [26]. The optimum dosage of fly ash is 0.3. When w/b = 0.4 and t = 28, 120, 540, and 1800 days, D_cf_ at m_f_ = 0.3 is smaller than that at m_f_ = 0.1 by 33.2%, 36.4%, 48.1%, and 69.7% for C_a_ = 0.55; by 22.6%, 26.9%, 49.0%, and 66.1% for C_a_ = 0.65; and by 33.4%, 36.7%, 38.7%, and 54.5% for C_a_ = 0.75, respectively. Similarly, when w/b = 0.5 and t is 28, 120, 540, and 1800 days, D_cf_ at m_f_ = 0.3 is smaller than that at m_f_ = 0.1 by 28.2%, 37.1%, 37.4%, and 46.4% for C_a_ = 0.55; by 22.6%, 36.2%, 46.7%, and 62.8% for C_a_ = 0.65; and by 33.0%, 38.3%, 52.4%, and 56.9% for C_a_ = 0.75, respectively, which indicates that D_cf_ decreases with the increase of m_f_. This can be explained as follows. On the one hand, the micro-aggregate effect of fly ash improves the density of fly ash cement paste and the pozzolanic products are more compact than calcium-silicate-hydrates formed from cement hydration [27]. On the other hand, the cations induced by the pozzolanic hydration reaction are distributed in the pore solution and cause the pore walls to be charged negatively, thus hindering the diffusion of chloride ions [28,29].

It can also be seen from Figure 2 and Figure 3 that the chloride diffusivity of fly ash concrete decreases quickly with increasing t for a given t smaller than 540 days, but almost remains unchanged for a given t larger than 540 days. When w/b = 0.4 and m_f_ = 0.1, 0.2, and 0.3, D_cf_ at t = 540 days is smaller than that at t = 28 days by 71.7%, 76.0%, and 78.1% for C_a_ = 0.55; by 71.0%, 80.9%, and 81.8% for C_a_ = 0.65; and by 80.0%, 82.2%, and 81.7% for C_a_ = 0.75, respectively. When w/b = 0.5 and m_f_ = 0.1, 0.2, and 0.3, D_cf_ at t = 540 days is smaller than that at t = 28 days by 84.6%, 85.2%, and 86.6% for C_a_ = 0.55; by 79.2%, 80.9%, and 85.7% for C_a_ = 0.65; and by 78.1%, 82.2%, and 84.5% for C_a_ = 0.75, respectively. This is due to the fact that the hydration of fly ash exhibits a time-delay characteristic [5].

## 3. Chloride Diffusivity of Fly Ash Concrete

In modeling fly ash concrete, aggregates are considered to be spherical. An ITZ with low strength and high porosity is formed between aggregates and the cement paste matrix due to the low packing efficiency of cement particles near aggregates. It was experimentally confirmed that addition of fly ash results in a denser and stronger ITZ, possibly due to the filling effect of small fly ash particles [30,31]. However, the chloride diffusivity of ITZ is still much larger than that of fly ash cement paste [31]. Therefore, it is appropriate to model the ITZ as an independent phase. At present, a more consistent point of view is accepted from the experimental results obtained by Scrivener and Nemati [32] based on electron microscopy, i.e., the ITZ thickness is almost independent of the aggregate size. Thus, fly ash concrete is composed of spherical aggregates, ITZs with equal thickness, and a fly ash cement paste. The aggregate size D is divided into N grades [D_j_, D_j+1_] (j = 1, 2, …, N) according to sieving analyses. If the cumulative distribution function for the aggregate is approximately linear piece-wise in each sub-domain, the probability density function p_N_(D) and the cumulative distribution function P_N_(D) in terms of the number of aggregates are [33]

(1) $$p_{N}(D)=\sum_{j=1}^{N}\frac{6[P_{V}(D_{j+1})-P_{V}(D_{j})]}{{\mu \pi N}_{V}(D_{j+1}-D_{j})D^{3}}[H(D-D_{j})-H(D-D_{j+1})]$$

(2) $$P_{N}(D)=\sum_{j=1}^{k-1}\frac{3[P_{V}(D_{j+1})-P_{V}(D_{j})](D_{j}+D_{j+1})}{{\mu \pi N}_{V}D_{j}^{2}D_{j+1}^{2}}+\frac{3[P_{V}(D_{k+1})-P_{V}(D_{k})](D^{2}-D_{k}^{2})}{{\mu \pi N}_{V}(D_{k+1}^{2}-D_{k}^{2})D_{k}^{2}D^{2}}$$

where $D_{k}\leq D\leq D_{k+1}$ and the Heaviside step function H(x) is defined as

(3) $$H(x)=\{\begin{array}{ll} 1, & x>0\\ 0.5, & x=0\\ 0, & x<0 \end{array}$$

The number of aggregates per unit volume of aggregate, $N_{V}$, is obtained as

(4) $$N_{V}=\sum_{j=1}^{N}\frac{3(D_{j}+D_{j+1})[P_{V}(D_{j+1})-P_{V}(D_{j})]}{{\mu \pi D}_{j}^{2}D_{j+1}^{2}}.$$

It is well known that ITZs will overlap when spherical aggregates are randomly distributed in concrete and, the larger the aggregate volume fraction is, the higher the overlap degree of ITZ will be. In order to consider the ITZ overlap and to construct a two-phase composite material model, composed of equivalent spherical aggregates and a fly ash cement paste, the following three-step scheme is adopted to determine the equivalent ITZ thickness.

1. Calculation of ITZ volume fraction f_i_ without ITZ overlap effect. If the equivalent ITZ thickness is denoted as h_eq_, the ITZ volume for a single spherical aggregate is given by

(5) $$V_{i}=\frac{\pi }{6}[{\left(D+2h_{\mathrm{eq}}\right)}^{3}-D^{3}]$$

Thus, the ITZ volume fraction f_i_ is equal to

(6) $$\begin{array}{l} f_{i}=\int_{D_{1}}^{D_{N+1}}\frac{6f_{a}}{\mu \pi \langle D^{3}\rangle }p_{N}(D)({\pi D}^{2}h_{\mathrm{eq}}+2{\pi \mathrm{Dh}}_{\mathrm{eq}}^{2}+4{\pi h}_{\mathrm{eq}}^{3}/3)\mathrm{dD}\\ \;=\frac{6f_{a}}{\mu \pi \langle D^{3}\rangle }(\pi \langle D^{2}\rangle h_{\mathrm{eq}}+2\pi \langle D\rangle h_{\mathrm{eq}}^{2}+4{\pi h}_{\mathrm{eq}}^{3}/3) \end{array}$$

where f_a_ is the aggregate volume fraction, and 〈D〉 and $\langle D^{2}\rangle$ are the first and second moments of area of p_N_(d), respectively. For any integer k, $\langle D^{k}\rangle$ is defined as

(7) $$\langle D^{k}\rangle =\int_{D_{1}}^{D_{N+1}}D^{k}p_{N}(D)\mathrm{dD}$$

Substitution of Equation (1) into Equation (7) yields

(8) $$\langle D^{k}\rangle =\{\begin{array}{l} \sum_{j=1}^{N}\frac{6[P_{V}(D_{j+1})-P_{V}(D_{j})]\ln(D_{j+1}/D_{j})}{{\pi \mu N}_{V}(D_{j+1}-D_{j})},\;k=2\\ \sum_{j=1}^{N}\frac{6[P_{V}(D_{j+1})-P_{V}(D_{j})](D_{j+1}^{k-2}-D_{j}^{k-2})}{\left(k-2){\pi \mu N}_{V}(D_{j+1}-D_{j}\right)},\;k\neq 2 \end{array}$$

2. Calculation of ITZ volume fraction f_i_ with ITZ overlap effect. Based on the geometric statistical theory of composite materials, Lu and Torquato [34] proposed a method for calculating the ITZ volume fraction, in which the overlap between adjacent ITZs is taken into account. According to their theory, f_i_ is given by

(9) $$f_{i}=(1-f_{a})[1-\exp(-t_{1}h-t_{2}h^{2}-t_{3}h^{3})]$$

where h is the practical ITZ thickness and t_1_, t_2_, and t_3_ are expressed in terms of f_a_ and $\langle D^{k}\rangle$ as

(10) $$t_{1}=\frac{6f_{a}\langle D^{2}\rangle }{(1-f_{a})\langle D^{3}\rangle }$$

(11) $$t_{2}=\frac{1{2f}_{a}\langle D\rangle }{(1-f_{a})\langle D^{3}\rangle }+\frac{18f_{a}^{2}{\langle D^{2}\rangle }^{2}}{{\left(1-f_{a}\right)}^{2}{\langle D^{3}\rangle }^{2}}$$

(12) $$t_{3}=\frac{{8f}_{a}}{(1-f_{a})\langle D^{3}\rangle }+\frac{2{4f}_{a}^{2}\langle D\rangle \langle D^{2}\rangle }{{\left(1-f_{a}\right)}^{2}{\langle D^{3}\rangle }^{2}}+\frac{{8f}_{a}^{3}{\langle D^{2}\rangle }^{3}\lambda }{{\left(1-f_{a}\right)}^{3}{\langle D^{3}\rangle }^{3}}$$

3. Determination of equivalent ITZ thickness h_eq_. From Equations (6) and (9), h_eq_ can be obtained as

(13) $$h_{\mathrm{eq}}=\sqrt[{3}]{-\frac{q}{2}+\sqrt{\frac{q^{2}}{4}+\frac{p^{3}}{27}}}+\sqrt[{3}]{-\frac{q}{2}-\sqrt{\frac{q^{2}}{4}+\frac{p^{3}}{27}}}-\langle D\rangle$$

where p and q are defined as

(14) $$p=3(\langle D^{2}\rangle -{\langle D\rangle }^{2})/4$$

(15) $$q=\frac{{\langle D\rangle }^{3}}{4}-\frac{3\langle D\rangle \langle D^{2}\rangle }{8}-\frac{3f_{i}}{4{\pi N}_{V}f_{a}}$$

As can be seen from Equations (13) to (15), the main factors that influence h_eq_ include f_a_, p_N_(D), and h.

With h_eq_ known, the chloride diffusivity of an equivalent spherical aggregate, composed of a spherical aggregate and an equivalent ITZ, is given by [35]

(16) $$D_{\mathrm{ea}}=D_{i}+D_{i}{\left[{\left(\frac{V_{a}}{V_{a}+V_{i}}\cdot \frac{D_{a}-D_{i}}{D_{i}+(D_{a}-D_{i})/3}\right)}^{-1}-\frac{1}{3}\right]}^{-1}$$

where D_a_ and D_i_ are the chloride diffusivities of aggregate and ITZ and V_a_ and V_i_ are the volumes of the aggregate and the ITZ, respectively. It is obvious that

(17) $$V_{a}=\frac{{\pi D}^{3}}{6}$$

(18) $$V_{i}=\frac{\pi [{\left(D+2h_{\mathrm{eq}}\right)}^{3}-D^{3}]}{6}.$$

Since the density of aggregate is much higher than those of ITZ and bulk paste, the chloride diffusivity D_a_ can be assumed to be zero. Thus, Equation (16) reduces to

(19) $$D_{\mathrm{ea}}=\frac{2V_{i}}{3V_{a}+2V_{i}}D_{i}$$

**Figure 4:**
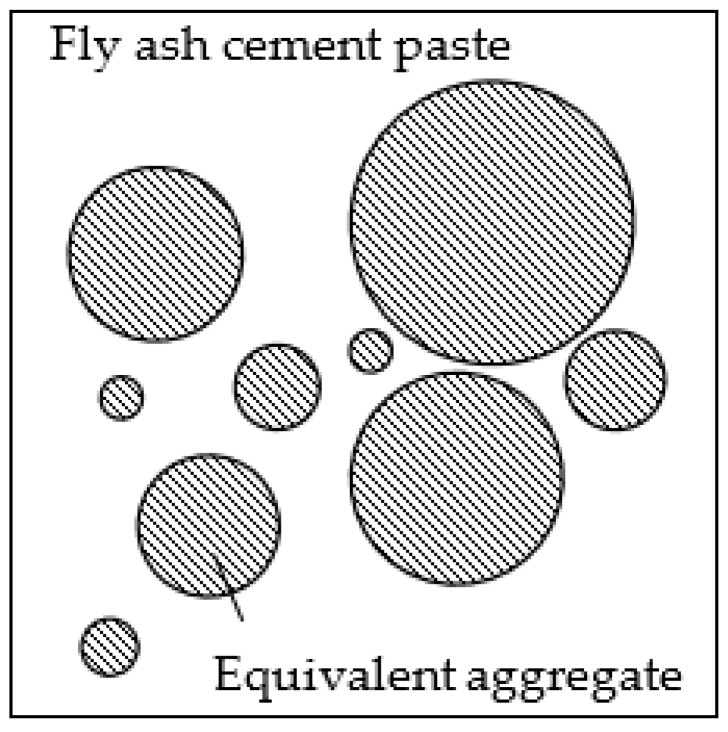
Two-phase fly ash concrete composed of fly ash cement paste and equivalent aggregates.

With the equivalent aggregate model, three-phase fly ash concrete can be reduced to a two-phase composite material, composed of equivalent aggregates of various sizes and chloride diffusivities encompassed by a fly ash cement paste of chloride diffusivity D_f_, as shown in Figure 4. According to the Maxwell method, the chloride diffusivity of a two-phase composite material is given by

(20) $$\frac{D_{e}-D_{1}}{D_{e}+{2D}_{1}}=\varphi_{2}\frac{D_{2}-D_{1}}{D_{2}+2D_{1}}$$

where D_e_, D_1_, and D_2_ are the chloride diffusivities of the effective medium, phase 1, and phase 2, respectively, and φ_2_ is the volume fraction of phase 2. By extending the Maxwell method to the fly ash concrete with N-type aggregates, the chloride diffusivity is given by

(21) $$\frac{D_{\mathrm{cf}}-D_{f}}{D_{\mathrm{cf}}+{2D}_{f}}=\sum_{j=1}^{N}\frac{{(D}_{\mathrm{ea},j}-D_{f})\varphi_{j}}{D_{\mathrm{ea},j}+2D_{f}}$$

where φ_j_ is defined as

(22) $$\varphi_{j}=\frac{f_{a}d_{\mathrm{ea},j}^{3}}{\sum_{j=1}^{N}d_{\mathrm{ea},j}^{3}}$$

with D_f_ and D_ea,j_ being the chloride diffusivities of the fly ash cement paste and the j-th equivalent aggregate, and d_ea,j_ and φ_j_ being the diameter and volume fraction of the j-th equivalent aggregate, respectively.

Finally, solving Equation (21) gives the chloride diffusivity of fly ash concrete

(23) $$D_{\mathrm{cf}}=D_{f}\frac{1+2\sum_{j=1}^{N}\varphi_{j}\frac{D_{\mathrm{ea},j}-D_{f}}{D_{\mathrm{ea},j}+2D_{f}}}{1-\sum_{j=1}^{N}\varphi_{j}\frac{D_{\mathrm{ea},j}-D_{f}}{D_{\mathrm{ea},j}+2D_{f}}}.$$

## 4. Experimental Verification and Discussions

Using the analytical method proposed by Zhou et al. [12], the chloride diffusivity D_f_ of fly ash cement paste can be estimated. To evaluate the chloride diffusivity of the equivalent aggregate from Equation (19), the ITZ chloride diffusivity D_i_ needs to be known beforehand. Since it is still difficult to measure the ITZ chloride diffusivity directly through testing, experimental calibration is conducted to determine the ratio D_i_/D_b_. For this purpose, the experimental results for w/b = 0.5 and C_a_ = 0.55, 0.65, and 0.75 are selected. As for the ITZ thickness, available experimental results do not achieve a consistent conclusion. Qian et al. [36] indicated that addition of fly ash decreases the ITZ thickness by 37%. However, other studies showed that the effect of fly ash on the ITZ thickness is insignificant [30,37,38]. In view of this, the ITZ thickness in fly ash concrete is assumed to be similar to that in normal concrete as a preliminary study. Since the ITZ thickness is usually in the range of 0.01 to 0.05 mm for normal concrete [39], h is taken as 0.03 mm. Thus, the ratio of D_i_/D_b_ at t = 28, 60, 120, 540, and 1800 days is back-calculated as 10.5, 10.8, 8.4, 5.08, and 1.8 for m_f_ = 0.1; 11.3, 11.7, 8.1, 2.86, and 1.35 for m_f_ = 0.2; and 12.6, 10.6, 9.47, 2.94, and 1.43 for m_f_ = 0.3, respectively. It is seen that D_i_/D_b_ is seldom influenced by the fly ash content but decreases with the increase of t, which agrees well with the results of Bentz [40]. The average ratio of D_i_/D_b_ at m_f_ = 0.1, 0.2, and 0.3 is equal to 11.46, 11.04, 8.65, 3.62, and 1.53 for t = 28, 60, 120, 540, and 1800 days, respectively. Through regression analysis, D_i_/D_b_ can be expressed as

(24) $$D_{i}/D_{b}=43t^{-0.366}$$

with a correlation coefficient of 0.912. Furthermore, when m_f_ = 0.1, 0.2, and 0.3, the correlation coefficient between the analytical method and the experimental results is calculated as 0.997, 0.998, and 0.996 for C_a_ = 0.55; 0.993, 0.995, and 0.994 for C_a_ = 0.65; and 0.996, 0.998, and 0.998 for C_a_ = 0.75, respectively.

With D_i_/D_b_ known, the chloride diffusivity of fly ash concrete can be evaluated for w/b = 0.4. The results are shown in Figure 2, which demonstrates that the analytical method is in good agreement with the experimental results. When m_f_ = 0.1, 0.2, and 0.3, the correlation coefficient between them is 0.986, 0.992, and 0.997 for C_a_ = 0.55; 0.979, 0.989, and 0.992 for C_a_ = 0.65; and 0.993, 0.985, and 0.982 for C_a_ = 0.75, respectively. Therefore, the validity of the analytical method is verified.

**Figure 5:**
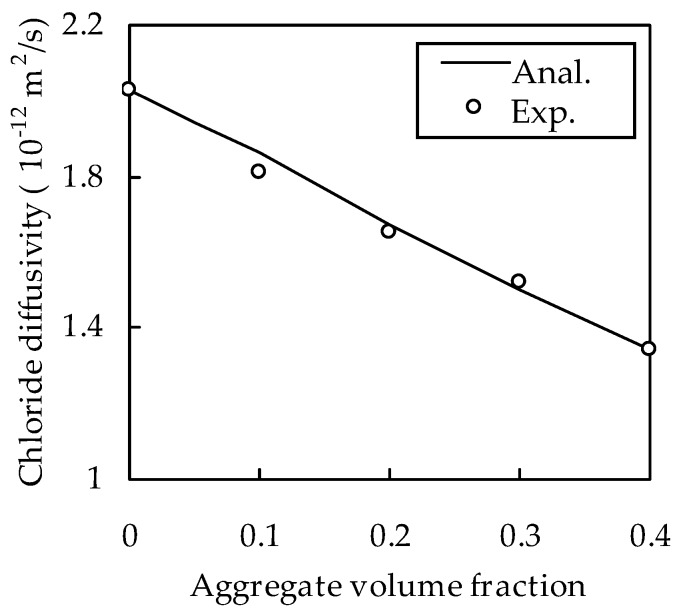
Comparison with experimental results of Yang and Su [41].

To further validate the analytical method, the test results of Yang and Su [41] were selected. In their test, an ordinary Portland cement of type ASTM I was used and the water/binder ratio was 0.4. The aggregate volume fraction was 0, 0.1, 0.2, 0.3, and 0.4, and 10% and 20% of cement by mass was replaced by fly ash or slag. The maximum and minimum aggregate diameters were 9.5 and 0.15 mm, respectively. For an aggregate grade at 0.15–0.3, 0.3–0.6, 0.6–1.18, 1.18–2.36, 2.36–4.75, 4.75–9.5, and 9.5–16 mm, the volume fraction was 2.83%, 8.57%, 28.9%, 27.8%, 23.5%, 8.1%, and 0.3%, respectively. The specimens were cast at room temperature. After 24 h of casting, they were taken out of molds and immediately immersed in a water tank with a curing temperature of 23 °C. After 12 months, the chloride diffusivity was measured by a migration method. The test results are shown in Figure 5. As in the last example, h = 0.03 mm. The chloride diffusivity of cement paste is equal to 2.03 × 10^−12^ m^2^/s and D_i_ is calibrated from the chloride diffusivity of concrete, i.e., 8.05 × 10^−12^ m^2^/s, measured at C_a_ = 0.4, as 1.34 × 10^−12^ m^2^/s. With these parameters, D_cf_ with different C_a_ can be calculated by the proposed analytical method; the results are shown in Figure 5. It can be seen from Figure 5 that the analytical method agrees well with the experimental results, with a correlation coefficient of 0.996. Therefore, the validity of the analytical method is further verified.

Although the proposed analytical method was preliminarily verified by the experimental results, more chloride diffusion tests of fly ash concrete and microstructural experiments need to be conducted to further validate the analytical method. Especially, the effects of fly ash content and water/binder ratio on the thickness, porosity, and chloride diffusivity of ITZ need to be investigated in future studies.

## 5. Conclusions

The chloride diffusivity of fly ash concrete has been measured through experiment and estimated based on the equivalent aggregate model and the generalized Maxwell method. The main conclusions of this study are as follows:

1. From the test results, it has been found that the chloride diffusivity of fly ash concrete decreases almost linearly with an increase in aggregate volume fraction. The optimum fly ash content is 0.3. The chloride diffusivity exhibits an evidently decreasing trend for a given curing time within 540 days, but almost remains unchanged for a given curing time from 540 to 1800 days.

2. An equivalent spherical aggregate model has been proposed and the equivalent ITZ thickness has been derived analytically. The main influential factors on the equivalent ITZ thickness include the aggregate gradation, the aggregate volume fraction, and the practical ITZ thickness. The chloride diffusivity of fly ash concrete has been estimated by the analytical method and verified against experimental results.

## Figures and Tables

**Table 1:** Chemical compositions of fly ash and cement.

Material	CaO (%)	SiO_2_ (%)	Al_2_O_3_ (%)	Fe_2_O_3_ (%)	MgO (%)	SO_3_ (%)	K_2_O (%)	Na_2_O (%)
Fly ash	5.40	47.00	26.37	5.23	0.38	0.85	0.67	0.61
Cement	65.31	21.21	3.88	3.25	1.12	0.98	0.57	0.12

**Table 2:** Mix proportions of fly ash concrete.

w/b	Dosage of Material (kg/m^3^)			
	Fly Ash	Cement	Water	Aggregate
0.4	61.7	555.3	246.8	1474.1
0.4	121.5	485.9	242.9	1474.0
0.4	179.4	418.6	239.2	1473.9
0.4	48.0	432.0	192.0	1742.0
0.4	91.9	367.6	183.8	1742.1
0.4	132.3	308.6	176.3	1742.0
0.4	34.3	308.5	137.1	2010.0
0.4	67.5	269.9	135.0	2010.0
0.4	99.7	232.5	132.9	2010.0
0.5	54.3	488.4	271.4	1474.0
0.5	107.0	428.1	267.6	1473.9
0.5	158.3	369.4	263.9	1474.0
0.5	42.2	379.9	211.0	1742.0
0.5	83.2	333.0	208.1	1741.9
0.5	123.1	287.3	205.2	1742.0
0.5	30.1	271.3	150.7	2010.0
0.5	59.5	237.8	148.6	2010.0
0.5	88.0	205.2	146.6	2010.0
